# Approaches and processes for paediatric chest X-ray classification used in the SHINE TB treatment-shortening trial

**DOI:** 10.5588/ijtld.24.0076

**Published:** 2024-11-01

**Authors:** M. Palmer, M.M. van der Zalm, H.S. Schaaf, P. Goussard, J. Morrison, J.A. Seddon, S. Hissar, D. Baskaran, A. Kinikar, P. Raichur, E. Wobudeya, C. Chabala, K. Lebeau, A.M. Crook, A. Turkova, D. Gibb, A.C. Hesseling

**Affiliations:** 1 Desmond Tutu TB Centre, Department of Paediatrics and Child Health, Stellenbosch University, Cape Town, South Africa; 2 Department of Paediatrics and Child Health, Stellenbosch University, Cape Town, South Africa; 3 Department of Infectious Disease, Imperial College London, London, United Kingdom; 4 National Institute for Research in Tuberculosis, Chennai, India; 5 B.J. Government Medical College, Pune, India; 6 Makerere University–Johns Hopkins University Research Collaboration, Kampala, Uganda; 7 University Teaching Hospital, Lusaka, Zambia; 8 Medical Research Council Clinical Trials Unit, University College London, London, United Kingdom

**Keywords:** chest X-ray, chest radiograph, methods, paediatric TB, SHINE

## Abstract

**Introduction:**

SHINE (Shorter Treatment for Minimal Tuberculosis in Children) was the first Phase 3 paediatric TB treatment shortening trial. Robust chest X-ray (CXR) classification methods were integral to excluding severe disease for trial eligibility and to retrospectively adjudicating TB status at baseline. We describe and critically evaluate the CXR classification approaches and processes used in the SHINE trial.

**Methods:**

Children with non-severe TB were randomised to 4- vs 6-months anti-TB treatment. Radiologically non-severe TB was defined on CXR. CXRs were systematically interpreted by on-site clinicians prospectively for eligibility determination and retrospectively by experts to inform adjudication of baseline TB status and disease severity.

**Results:**

A screening CXR was successfully obtained from all 1,204 enrolled children; 1,134 CXRs from children with intra-thoracic TB were reviewed by expert readers. Compared with the expert panel, enrolling clinicians classified more CXRs as abnormal and ‘typical TB’ and all as radiologically non-severe. The expert panel retrospectively classified 71/1,134 (6%) CXRs as severe. Of these, 4 (5.6%) had unfavourable outcomes compared with 34 (3.0%) in the trial overall.

**Discussion:**

Using CXRs to classify radiological disease severity and inform eligibility decisions in real-time by local enrolling clinicians was feasible and safe in this large paediatric TB trial. Retrospective central expert CXR review was successful. Refinement of the CXR methods for the classification of both disease severity and TB status could support standardised implementation in routine care and research.

## RéSUMé

**Introduction:**

SHINE (Shorter Treatment for Minimal Tuberculosis in Children) a été le premier essai de phase 3 sur le raccourcissement du traitement de la TB pédiatrique. Des méthodes robustes de classification par radiographie thoracique (CXR) ont joué un rôle essentiel dans l'exclusion de la maladie grave pour l'admissibilité à l'essai et dans l'évaluation rétrospective du statut de la TB au départ. Nous décrivons et évaluons de manière critique les approches et les processus de classification CXR utilisés dans l'essai SHINE

**Méthodes:**

Les enfants atteints de TB non sévère ont été randomisés pour recevoir un traitement antituberculeux de 4 contre 6 mois. La TB radiologiquement non sévère a été définie sur la radiographie thoracique. Les radiographies thoraciques ont été systématiquement interprétées par les cliniciens sur place de manière prospective pour déterminer l'admissibilité et rétrospectivement par des experts pour éclairer l'évaluation de l'état de base de la TB et de la gravité de la maladie.

**Résultats:**

Une radiographie thoracique de dépistage a été obtenue avec succès chez les 1 204 enfants inscrits ; 1 134 radiofréquences radiophoniques d'enfants atteints de TB intrathoracique ont été examinées par des lecteurs experts. Par rapport au panel d'experts, les cliniciens recrutés ont classé davantage de radiofréquences comme anormales et « TB typiques » et toutes comme radiologiquement non graves. Le groupe d'experts a classé rétrospectivement 71/1 134 (6%) RCP comme étant graves. Parmi ceux-ci, 4 (5,6%) ont eu des résultats défavorables, contre 34 (3,0%) dans l'ensemble de l'essai.

**Discussion:**

L'utilisation de la radiographie thoracique pour classer la gravité de la maladie radiologique et éclairer les décisions d'éligibilité en temps réel par les cliniciens locaux recrutés était réalisable et sûre dans cet essai de grande envergure sur la TB pédiatrique. L'examen radiophonique central a été couronné de succès. L'affinement des méthodes CXR pour la classification de la gravité de la maladie et du statut de la TB pourrait soutenir une mise en oeuvre standardisée dans les soins de routine et la recherche.

Classifying TB disease with a high degree of certainty and characterising the disease spectrum (including severity) are integral methodological components of paediatric TB trials. In phase 3 clinical trials investigating new anti-TB drugs and regimens, accurate adjudication of TB status and disease spectrum at enrolment impacts the interpretation of treatment outcomes and trial end-points. Diagnostic certainty of TB disease is more challenging in young children, given that the disease is typically paucibacillary with low microbiological confirmation rates.^1–3^ Disease severity classification is also more challenging in children as microbiological markers of severe disease (such as smear positivity or shorter culture time-to-positivity) are not optimal in paucibacillary disease, and there is an absence of empirical data identifying risk factors for ‘severe disease’. Consequently, there is a reliance on chest X-ray (CXR) within the context of clinical case definitions to allocate TB status and disease spectrum in children.^4,5^

Consensus clinical case definitions for paediatric TB diagnosis, updated in 2015 and assigned retrospectively, classify children as having ‘confirmed’, ‘unconfirmed’ or ‘unlikely’ intra-thoracic TB using standard criteria and allow for meaningful comparison of results between studies.^4,5^ A key criterion that can differentiate ‘unconfirmed TB’ from ‘unlikely’ TB is the CXR findings. The recommended methodology for CXR interpretation in these case definitions is that CXRs are interpreted by two independent, blinded, appropriately qualified and experienced readers, with a third reader arbitrating discordant reads, and categorised as ‘consistent with TB’ or not, using a standardised approach and pre-determined terminology.^4,5^ While these guidelines are important and support uniformity in the CXR reading processes between studies, they were developed by an expert panel who agreed on them by a narrow margin and experience with the feasibility of their practical implementation in a large paediatric TB clinical trial has not been described.^6,7^ Also, the process used to classify radiological disease severity is not included in the scope of these case definitions.

The ‘Shorter Treatment for Minimal Tuberculosis in Children’ (SHINE, ISRCTN63579542) trial was a phase 3, randomised open, TB treatment shortening trial which demonstrated that a 4-month treatment regimen using first-line TB drugs at standard doses was non-inferior to the standard-of-care 6-month regimen in children with non-severe, intra-thoracic TB or peripheral TB lymphadenitis.^8,9^ CXR interpretation was fundamental to the conduct of the trial for both the real-time determination of participant eligibility by the enrolling on-site clinicians and the retrospective CXR classification by the expert panel, which informed the assignment of baseline TB status, an important variable in the secondary efficacy analyses. In this paper, we describe the role of the CXR in the SHINE trial and critically evaluate the methods used to classify the screening CXRs of enrolled children by the on-site clinicians and the central expert panel.

## Methods

### Trial procedures

**Figure 1:**
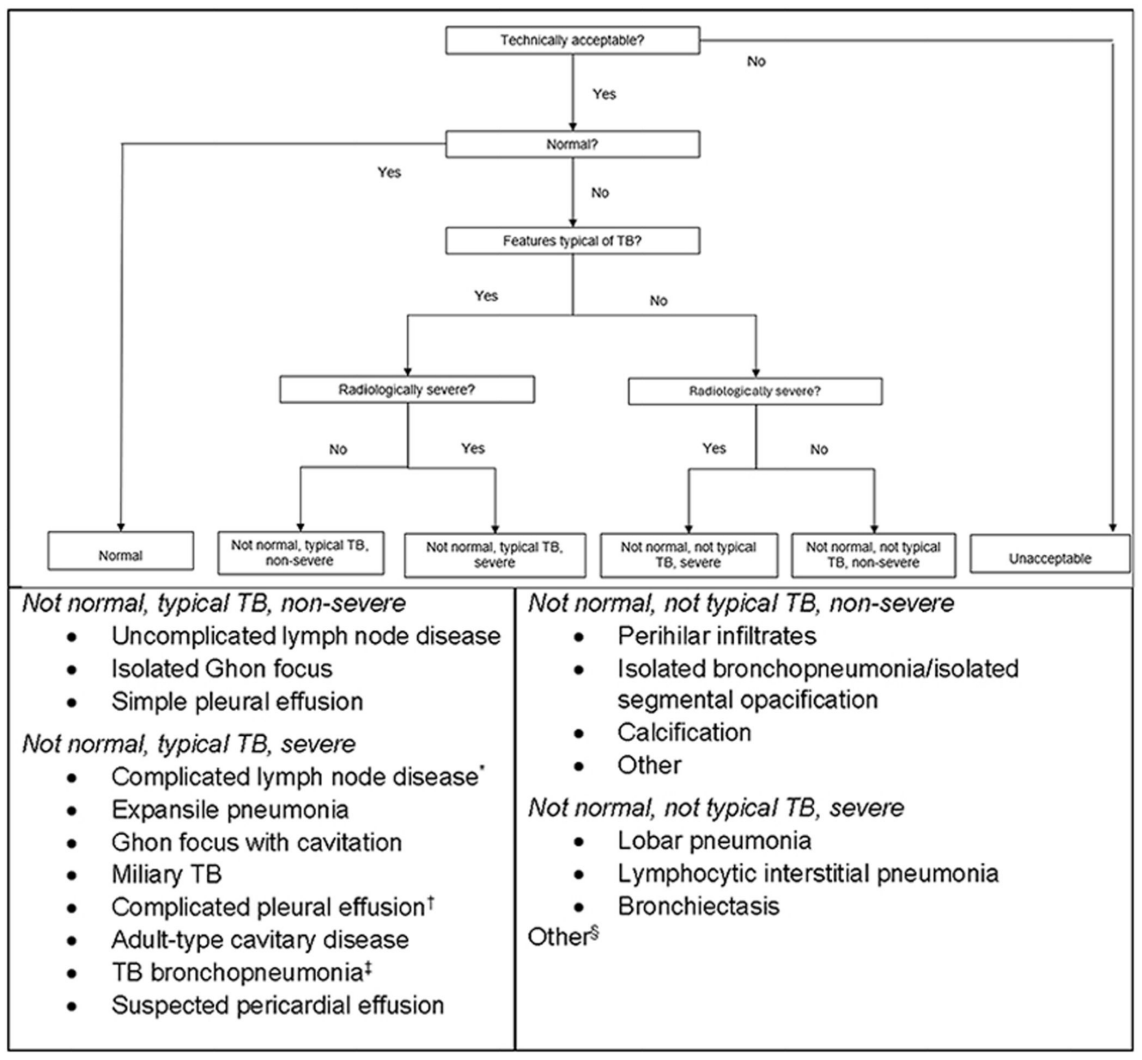
Schematic illustration of the process used by on-site clinicians and expert readers to classify each individual chest x-ray according to radiological pattern in the SHINE trial Schematic illustration of the process used by on-site clinicians and expert readers to classify each chest X-ray by radiological pattern in the SHINE trial. *Lymph node enlargement with bilateral airway compression or associated with unilateral hyperinflation or collapse or ≥1 lobe opacification (consolidation). ^†^Pleural effusion with pneumothorax or loculated or with underlying opacification (consolidation). ^‡^Bronchopneumonic consolidation with cavities and presence of enlarged lymph nodes. ^§^Included multi-lobar pneumonia. SHINE = Shorter Treatment for Minimal Tuberculosis in Children.

Children were eligible for enrolment into SHINE at five sites in India, Uganda, Zambia and South Africa if they were <16 years old with newly diagnosed, presumed drug-susceptible, peripheral TB lymphadenitis and/or non-severe intra-thoracic TB that was smear-negative for acid-fast bacilli (AFB) on respiratory samples.^8,9^ The radiological classification of non-severe TB on CXR included uncomplicated lymph node disease and/or opacification of <1 lobe and/or pleural effusion classified as simple (Figure 1). In the absence of empirical data to inform radiological disease severity classification in children, this approach was based on a conceptual understanding of the pathophysiology of paediatric TB and expert opinion.^10–12^ The primary efficacy outcome of the trial was unfavourable status (defined elsewhere) by Week 72.^8,9^

The trial was approved by all relevant national and local ethics committees, regulatory authorities and the University College London (UCL) Health Research Ethics Committee, London, UK. Children were enrolled after obtaining written informed consent from the parents/legal guardians and assent from children ≥7 years old if appropriate.

### CXR timing and reading overview

CXRs were taken at the screening visit (before enrolment), at Week 24, and at other time points if clinically indicated (including if TB treatment failure or TB recurrence was suspected). The methods and results outlined in this paper relate only to the screening CXRs from children who were subsequently enrolled on the trial with intra-thoracic TB (those with peripheral TB lymphadenitis without intra-thoracic TB are excluded). The type of CXR taken—anteroposterior (AP) or posteroanterior (PA)—varied with the child’s age, and lateral films were not consistently available across all sites. The imaging equipment used, whether digital or analogue, depended on the resources at each site. Analog CXRs were digitised using a camera (per standard operating procedure) or a CXR film scanner.

CXRs were interpreted using two independent processes: 1) review on-site in real-time by a local study clinician and 2) review retrospectively by a central panel of independent experts blinded to clinical, site and treatment information and each other’s reads. CXR data from on-site clinicians and members of the expert panel were captured on complementary standard case report forms. Each CXR was systematically evaluated and independently classified for 1) technical acceptability and, if technically acceptable, for 2) radiological diagnostic certainty (normal, abnormal ‘typical TB’ or abnormal ‘not typical TB’, and 3) disease severity. This approach classified CXRs according to radiological patterns considered ‘typical’ and ‘not typical’ of TB and ‘severe’ and ‘not severe’ (Figure 1). Understanding that not all children diagnosed with TB will have ‘typical’ CXR features, radiological disease severity was determined for all CXRs irrespective of whether the CXR was classified as being ‘typical TB’ or ‘not typical TB’.

### On-site CXR review

CXRs were reviewed in real-time by on-site clinicians to verify TB diagnosis and exclude radiologically severe intra-thoracic disease and thus inform eligibility. On-site clinicians had access to all information about the child’s TB exposure history, HIV status, clinical presentation and results from TB microbiological investigations. Before the study started, on-site clinicians attended an in-person interactive CXR interpretation training session. This session focused on the systematic assessment of technical acceptability and the classification of radiological diagnostic certainty and disease severity using the terminology and approach outlined in Figure 1. Each site received the training slides from the session and a printed booklet with examples of CXR images. A refresher training session was held virtually approximately one year into the trial.

### Retrospective central CXR review

**Figure 2:**
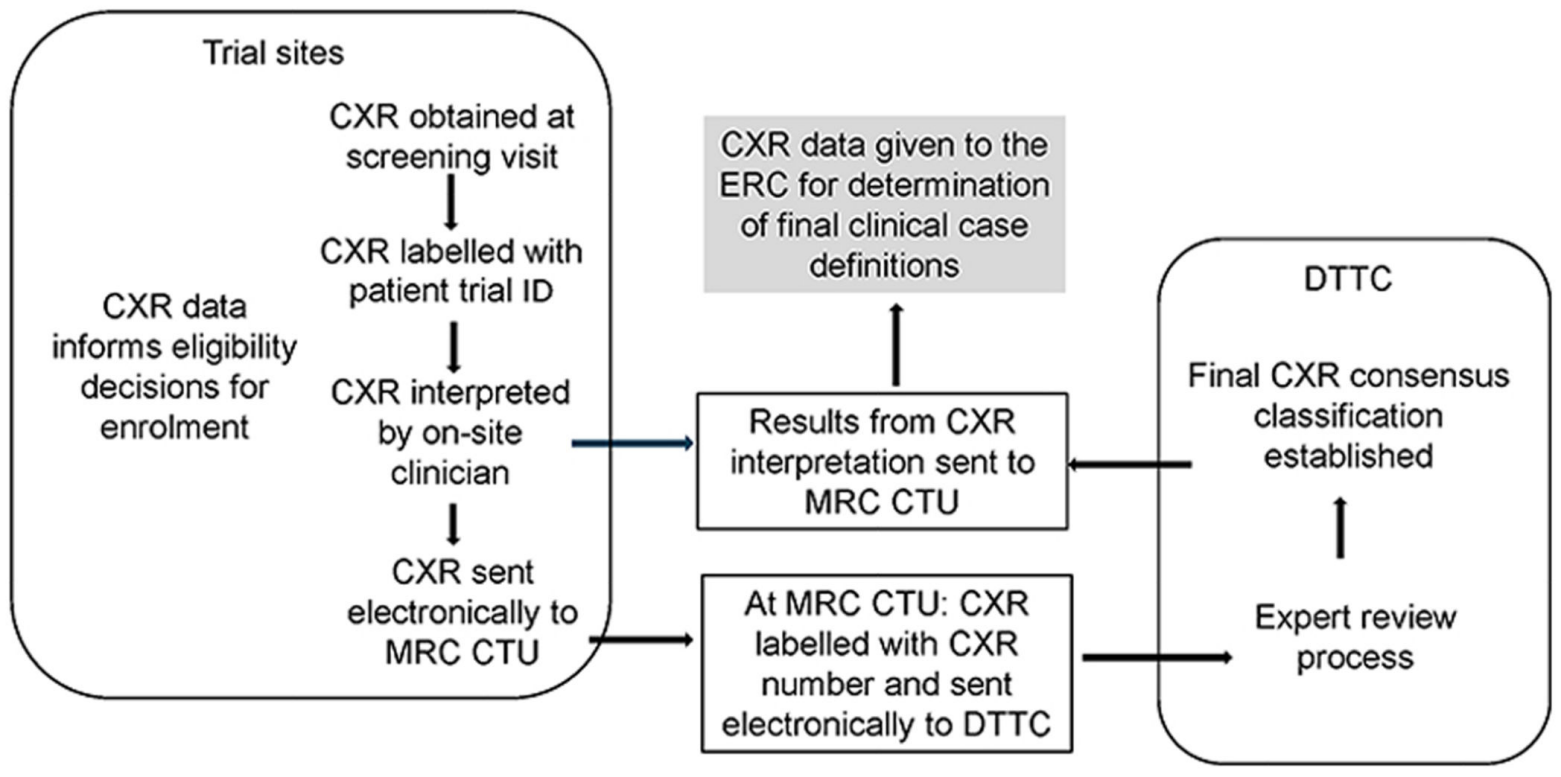
Flow of screening chest x-ray images and chest x-ray data between trial sites, central Medical Research Council Clinical Trials Unit and the Desmond Tutu TB Centre for expert central review Flow of screening CXR images and CXR data between trial sites, MRC CTU and the DTTC expert central review. CXR = chest X-ray; ERC = Expert Review Committee; MRC CTU = Medical Research Council Clinical Trials Unit; DTTC = Desmond Tutu TB Centre; SOE = schedule of evaluations; SHINE = Shorter Treatment for Minimal Tuberculosis in Children.

The retrospective CXR expert review was undertaken by the Desmond Tutu TB Centre (DTTC) at Stellenbosch University (SU) in Cape Town, South Africa. The CXR reading team was independent of the Clinical Trials Unit (CTU) at UCL, the trial sponsor. Screening CXRs were digitised, de-identified, and labelled using unique study participant trial numbers at each site, and they were electronically transferred to CTU using a secure file transfer portal. At CTU, each CXR was de-identified of the participant trial number and site details and relabelled with a CXR number before being electronically transferred for expert review (Figure 2). The CXR expert review panel was blinded to all participant information, including the enrolling site and only received screening CXRs from children who were subsequently enrolled on the trial (this excluded CXRs from 257 children who were screened but not enrolled).

**Figure 3:**
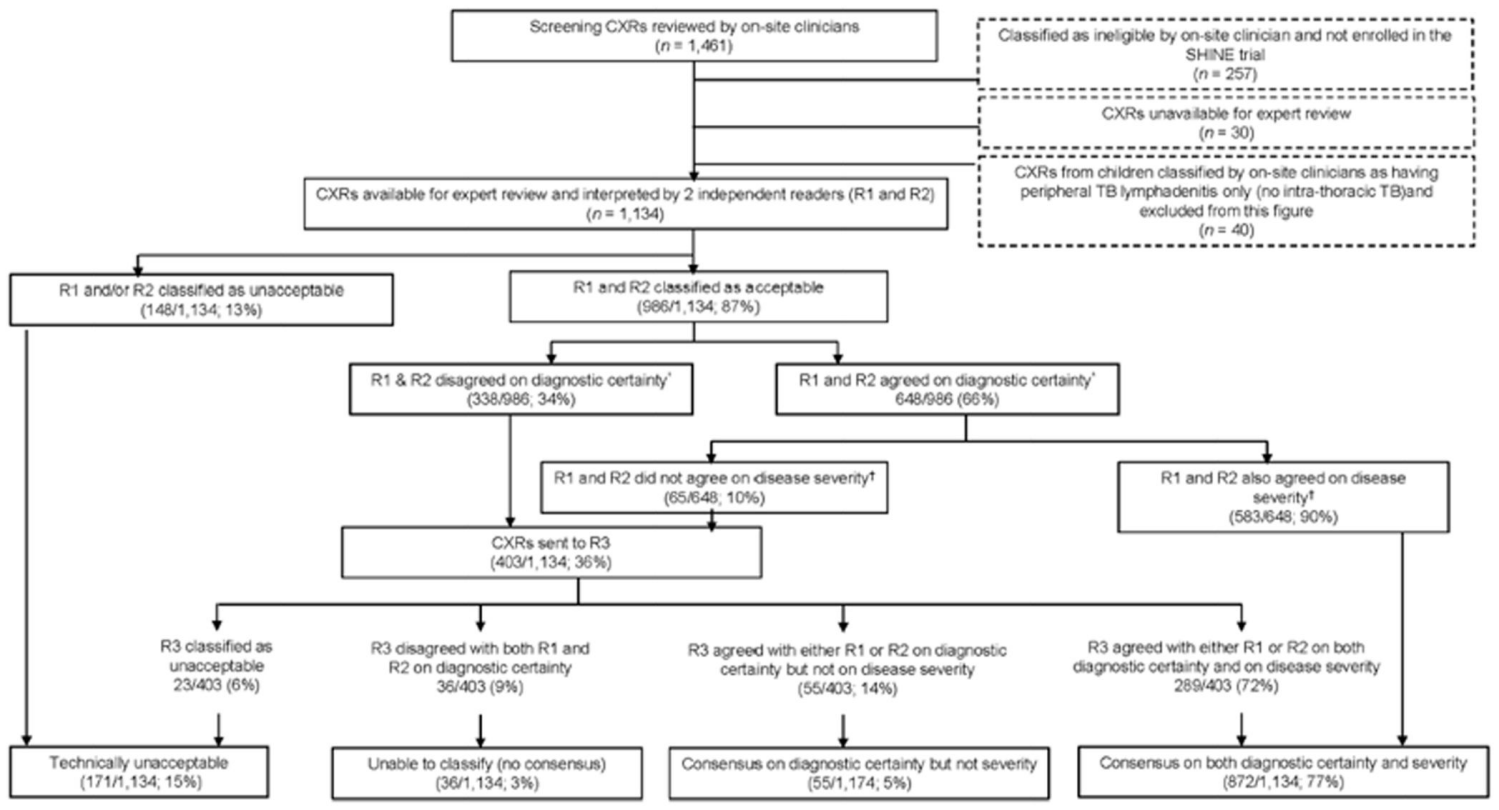
Process used to establish the final classification of screening chest x-rays from enrolled children after review by the expert reading panel FINAL CXR classification after expert review. Process used to establish consensus among expert readers on the classification of screening chest X-rays from children enrolled in the SHINE trial. SHINE = Shorter Treatment for Minimal Tuberculosis in Children; CXR = chest X-ray; R1 = expert reader who interpreted the first CXR; R2 = expert reader who interpreted the second CXR; R3 = expert reader who interpreted the third CXR; *’Diagnostic certainty’ was defined as normal (included cases where one reader classified the CXR as normal and one reader noted the presence of perihilar infiltrates/generalised hyperinflation only) OR abnormal ‘typical TB’ (see Figure 1 for definition) OR abnormal ‘not typical TB’ (defined in Figure 1). ^†^’Disease severity’, defined as radiologically severe OR non-severe (see Figure 1 for definition).

There were 4 CXR expert readers at DTTC: 1 paediatric TB specialist and three paediatric pulmonologists. Readers were not involved in the clinical management of any study participants. Every CXR was independently read by ≥2 expert readers and classified using the approach outlined in Figure 1. CXRs were classified as technically unacceptable if one or both of the first two readers classified them as such. If the two readers who interpreted a particular CXR disagreed on either radiological diagnostic certainty or disease severity, a third reader independently reviewed the CXR. The majority read was considered final (Figure 3). This final CXR classification was used by an independent clinical Expert Review Committee to adjudicate TB status at baseline using the consensus clinical case definitions;^1,2^ the key secondary efficacy trial outcomes were analysed only in the population of participants who were adjudicated by this committee to have TB (‘confirmed’ or ‘unconfirmed’) at baseline.

## Results

A total of 1,461 children were screened for the SHINE trial, and 1,204 were enrolled. Screening CXRs were successfully obtained from all and reviewed in real time by on-site clinicians. Of the 1,204 CXRs from enrolled children, 1,174 (98%) were available centrally for expert review (30 CXRs could not be located by sites), and 1,134 were from children classified as having intrathoracic TB by on-site clinicians (40 children were enrolled with peripheral TB lymphadenitis only) (Figure 3). On-site clinicians classified all CXRs as technically acceptable and as radiologically non-severe TB, while 171/1,134 (15%) CXRs were retrospectively classified as unacceptable and 71/1,134 (6%) as radiologically severe after the expert review process (Table 1). Of the 71 children with CXRs classified as severe by the expert panel, 4 (5.6%) had unfavourable outcomes — 1 death and 1 TB recurrence in each treatment arm. By comparison, 34 children (3.0%) had unfavourable outcomes in the trial overall.^9^ Onsite clinicians classified more CXRs as abnormal and ‘typical TB’ (1093 abnormal, 692 ‘typical TB’) than the expert panel (554 abnormal, 342 ‘typical TB’).

Of the 1,134 CXRs reviewed by the expert panel, 403 (36%) required review by a third reader for the final classification of either radiological diagnostic certainty (*n* = 338) or disease severity (*n* = 65). Overall, after review by two or three readers, there was agreement on radiological diagnostic certainty and disease severity for 872/1,134 (77%) (Figure 3). Inter-reader agreement between the expert CXR readers was fair for the assessment of all of the following: technical acceptability (j 0.25, 95% CI 0.20–0.30), normal vs. abnormal (κ 0.33, 95% CI 0.28–0.38), ‘typical TB’ vs. ‘not typical TB’ (κ 0.38, 95% CI 0.33–0.43) and ‘severe’ vs. ‘non-severe’ (κ 0.38, 95% CI 0.33–0.43). (Table 2).

## Discussion

SHINE was the first-ever phase-3 paediatric TB treatment shortening trial, and the results informed World Health Organization (WHO) guidelines, which now recommend a 4-month treatment regimen of standard first-line TB drugs for children with non-severe presumed/confirmed drug-susceptible TB disease.^9,13,14^ Robust CXR classification methods were integral to this trial. The trial demonstrated that clinical sites across diverse, high TB burden settings could obtain and interpret CXRs from children for the real-time determination of trial eligibility and that it was feasible to transfer CXRs centrally and to conduct an expert CXR review process.

The approach to disease severity classification on the SHINE trial was conceptualised to be scientifically robust but also sufficiently pragmatic to implement. The overall trial results reflect that this was successful. Only 71 children were ‘misclassified’ as having radiologically non-severe disease by the on-site clinicians, and only 4/71 had unfavourable outcomes; with 2 of these 4 in each treatment arm, it is unlikely that their unfavourable outcomes were related to the ‘misclassification’ and duration of TB treatment received. This supports the SHINE approach to disease severity stratification being safe, which is reassuring for the operationalisation of treatment shortening within routine care TB programmes, where CXR interpretation and disease severity decisions will primarily be made by non-expert clinicians.

The expert CXR reading panel only interpreted the screening CXRs from enrolled children. Of the 257 children screened but not enrolled, 144 were considered ineligible based on the on-site clinician assessment of severe TB disease. Interpretation of these 144 CXRs by the expert panel would have provided helpful insight into the on-site clinician’s assessment of radiological disease severity. The absence of this data is an acknowledged limitation of this analysis. While our expert reading panel did not include radiologists, we are confident in the quality of their radiological classification, given their specific experience with paediatric CXR interpretation in a high TB-burden setting.

The on-site clinicians classified more CXRs as abnormal and ‘typical TB’ than the blinded expert panel. This is likely because, in contrast to the experts, they were aware of the participant’s clinical risk factors, which would have influenced their interpretation of the radiological certainty of TB disease. Additionally, there were distinct differences in the experience and expertise between on-site clinicians and the expert readers and the final expert panel classification presented in Table 1 was established after review by >1 person.

While the recommended standardised approach to CXR classification for determining TB status in paediatric TB research is useful, the experience from SHINE is that further refinement could improve consistency in interpretation and implementation. Standard criteria for categorising technically acceptable CXR images (including lateral films) should be included, and we propose that interpreting the technical quality of paediatric CXRs should be a specific training focus area during the study set-up of future trials. The process of establishing consensus among readers requires elaboration. In the SHINE trial, the majority read after three reads were considered final; an alternative approach, which perhaps approximates true consensus more closely but is more resource-intensive, is to conduct consensus discussions between readers. Notably, the criteria for classifying a CXR as ‘consistent with TB’ (the terminology used in the consensus clinical case definitions) requires standardisation, particularly regarding CXR features such as isolated air-space disease (consolidation) and pleural effusion (classified as ‘not typical TB’ and ‘typical TB’ on the SHINE trial, respectively), which are more contentious and not currently consistently classified between studies.

The central expert review process in the SHINE trial was resource-intensive. Human resources were required for image management at all study sites, at the sponsor level and the central expert review site, in addition to the human resources required for the actual CXR reading. Access to specialist expert readers requires resources which are not always available in high TB-burden settings and may limit the feasibility of implementing these CXR classification methods in paediatric TB studies in the settings where they are most relevant.

Refining the recommended methods for classifying radiological certainty of TB disease on CXR and including a harmonised approach to radiological disease severity stratification in these guidelines could simplify their implementation, improve standardisation and support the operationalisation of the WHO recommendation on treatment shortening. However, there are innate limitations to CXR as a diagnostic imaging tool, which our results reflect and require acknowledgement. Concordance between all groups of CXR readers was suboptimal in the SHINE trial – this aligns with results from other studies which report interreader agreement for the classification of CXRs in the context of paediatric TB.^15–19^ CXR is a two-dimensional representation of three-dimensional space, there are challenges with the acquisition of good quality films in young children and subjectivity in interpretation, all of which contribute to CXR having lower overall diagnostic accuracy than advanced imaging modalities.

Despite the CXR’s limitations, it remains the most accessible imaging tool, and the SHINE trial demonstrated that CXR could be used for real-time stratification of radiological disease severity by on-site clinicians to facilitate appropriate treatment-duration selection. SHINE also showed that the currently recommended CXR classification methods for determining TB status by an expert panel could be successfully implemented in a large multi-country paediatric TB trial. Computer-aided detection (CAD) for the interpretation of CXRs for paediatric TB could reduce the inter-human variability in CXR interpretation, improve the efficiency of the CXR interpretation process and potentially provide global access to ‘expert’ CXR interpretation in resource-constrained settings. There is an urgent need to support TB programmes globally to implement disease severity stratification to facilitate access to the WHO-recommended 4-month treatment regimen for children with non-severe TB.

## Figures and Tables

**Table 1:** Final expert consensus read compared with on-site clinician read, screening CXR from children enrolled with intra-thoracic TB (*n* = 1,134*).

Final expert consensus read	On-site clinician read			
	Normal *n* (%)	Abnormal, typical TB, not severe *n*	Abnormal, not typical TB, not severe *n*	Total *n* (%)
Unacceptable^†^	5	98	68	171 (15)
Normal^‡^	31	196	146	373 (33)
Abnormal, typical TB, not severe	1	202	60	263 (23)
Abnormal, typical TB, severe	0	29	20	49 (4)
Abnormal, typical TB, no consensus severity^§^	1	22	7	30 (3)
Abnormal, not typical TB, not severe	3	88	74	165 (15)
Abnormal, not typical TB, severe	0	16	6	22 (2)
Abnormal, not typical TB, no consensus severity^§^	0	17	8	25 (2)
Unable to reach consensus^¶^	0	24	12	36 (3)
Total, *n* (%)	41 (4)	692 (61)	401 (35)	1,134

* Children classified at enrolment by on-site clinicians as having peripheral TB lymphadenitis only (with no intra-thoracic involvement) are excluded (*n* = 40).

† Technically unacceptable if one or both readers are classified as unacceptable.

‡ Normal includes cases where one reader classified as normal and one reader noted the presence of perihilar infiltrates/generalised hyperinflation only (158/373, 42%) OR when both readers classified as normal (215/373, 58%).

§ 2 readers agreed on radiological diagnostic certainty but not on radiological severity.

¶ No agreement between 2 of 3 readers on radiological diagnostic certainty.

CXR = chest X-ray.

**Table 2:** Inter-reader agreement on chest X-ray classification between expert readers* in the SHINE trial (*n* = 1,134).

	Agreement %	κ (95% CI)^†^
Agreement on acceptable vs not acceptable	86.9	0.25 (0.20–0.30)
Agreement on normal vs abnormal	68.5	0.33 (0.28–0.38)
Agreement on ‘typical TB’ vs ‘not typical TB’^‡^	68.8	0.38 (0.33–0.43)
Agreement on severe disease vs non-severe disease^§^	84.9	0.37 (0.33–0.43)

* Non-unique raters (i.e., Reader 1, Reader 2 and Reader 3 are not consistently the same person).

† Interpretation of κ co-efficient: 0.01–0.2 slight agreement, 0.21–0.4 fair agreement, 0.41–0.6 moderate agreement, 0.61–0.8 substantial agreement, 0.81–1.00 almost perfect agreement.

‡ CXRs classified as ‘normal’ were included as ‘not typical TB’ in this calculation.

§ CXRs classified as ‘normal’ were included as ‘non-severe’ in this calculation.

CI = confidence interval; CXR = chest X-ray.
